# Measuring Rotavirus Vaccine Impact in Sub-Saharan Africa

**DOI:** 10.1093/cid/ciz918

**Published:** 2019-09-23

**Authors:** A Duncan Steele, Michelle J Groome

**Affiliations:** 1 Enteric and Diarrheal Disease, Bill & Melinda Gates Foundation, Seattle, Washington, USA; 2 Respiratory and Meningeal Pathogens Research Unit, South African Medical Research Council, Johannesburg, South Africa; 3 Faculty of Health Sciences, University of the Witwatersrand, Johannesburg, South Africa

(See the Major Article by Khagayi, et al on pages 2298–305, and the Major Article by Otieno, et al on pages 2306–13.)

In July 2019, more than 300 scientists, researchers and public health officials gathered in Johannesburg, South Africa, for the 12th African Rotavirus Symposium to discuss the burden of rotavirus disease in Africa and the impact of rotavirus vaccination in countries where the vaccine has been introduced. The meeting was infused with optimism at the successful introduction of rotavirus vaccines in about 100 countries globally and the benefits that have been shown in terms of vaccine effectiveness and reductions in rotavirus hospitalizations in multiple countries, including in Africa [1]. Nevertheless, the African continent still faces challenges within this framework of success—several countries with large birth cohorts and high disease burden have not yet introduced the vaccine; global supply issues forced 4 countries using RotaTeq (Merck) to switch to a different product with relative urgency in late 2018 or early 2019 and contributed to delays in new introductions over the past 12 months, and several countries transitioning from Gavi support have expressed concerns about continuing full immunization programs when they must carry all associated costs, including costs of the vaccine.

Globally, rotavirus disease is estimated to cause the death of 185 390 (95% uncertainty interval [UI], 145 565–224 346) children <5 years of age every year [2]. Sub-Saharan Africa bears the brunt of these deaths and is reported to have a rotavirus mortality rate of 66.9/100,000 population <5 years old (95% UI, 53.3–82.3) compared with a global rate of 20.3/100 000 population (16.5–24.6) [3]. Thus, sub-Saharan Africa carries >80% of the global rotavirus mortality with only 8 countries bearing approximately 80% of that burden (Nigeria, Democratic Republic of the Congo, Niger, Chad, Burkina Faso, Core d’Ivoire, Ethiopia, and Uganda) [3]. Currently, 35 countries in sub-Saharan Africa have introduced rotavirus vaccines including 26 with support from Gavi, the Vaccine Alliance. In addition, several are approved for Gavi support and are planning introduction over the next 18 months. Unfortunately, delays in national introduction occurred owing to the global supply issue [4], which affected Democratic Republic of the Congo and Nigeria, countries with the largest birth cohorts.

The significance of these delays is demonstrated in the recent modeled estimates of the impact of rotavirus vaccine in Africa [5]. In 29 countries that had implemented the vaccines before December 2014, an estimated 21 000 deaths and >130 000 rotavirus hospitalizations were prevented in 2016 alone. If all African countries had been using the vaccines, an additional 47 000 deaths and >275 000 hospitalizations could have been prevented that year. Furthermore, an important study in Malawi demonstrated the population impact of rotavirus vaccine on all-cause diarrheal mortality rates, with reductions of approximately 31% at the community level (95% confidence interval, 1%–52%), highlighting the real-world benefits of vaccination in countries with high disease burden [6]. The delayed introductions carry a heavy penalty for African infants, and it is imperative that sub-Saharan Africa maintains the momentum of new country introductions and improves coverage of the rotavirus vaccines in countries where introduction has already taken place, to improve the outcomes in young children born on the continent.

Kenya was an early adopter of rotavirus vaccine in Africa, introducing the 2-dose, monovalent human rotavirus vaccine, Rotarix (GSK Biologicals) in July 2014. Long-term ongoing population-based surveillance in 2 regions of the country allowed observational studies of the vaccine [7, 8]. These 2 studies, conducted by the same research team and in the same demographic health surveillance areas in Kilifi on the coast and Siaya near Lake Victoria, used different methods to evaluate the effectiveness and impact of the introduction of rotavirus vaccine.

The case-negative case-control study design has been used in many studies conducted in different economic settings, including sub-Saharan Africa, to measure rotavirus vaccine effectiveness, and this approach has been shown to be an efficient and cost-effective alternative to the traditional case-control design [9]. Khagayi et al [7] used this approach and in so doing were able to take advantage of existing rotavirus surveillance programs started well before vaccine introduction. The vaccination status of rotavirus-positive case patients, identified through a qualitative rotavirus enzyme immunoassay, were compared with that of rotavirus-negative controls, who were enrolled through the same surveillance system used to identify the case patients. 

Case-control studies do present some challenges, however; for example, it is necessary to conduct the study when coverage rates are still relatively low. High coverage rates can be problematic, as evidenced in the study by Khagayi et al [7], in which 100% of the case patients were vaccinated in a single site and it was not possible to obtain an effectiveness estimate from that site. There is also often difficulty in establishing vaccination status for all enrolled children in African studies. However, because vaccination status is usually ascertained without knowledge of the case-control status, any inaccuracies in collection of vaccination status are unlikely to have biased the results. Despite these limitations, case-control studies provide valuable estimates of real-world effectiveness of the vaccine after routine introduction. Estimates for vaccine effectiveness of 2 doses of Rotarix vaccine against rotavirus hospitalization in Kenya were similar to those obtained from other sub-Saharan countries [10–12] and add to the growing body of evidence supporting continued use of these vaccines in Africa.

Of note, Khagayi et al [7] did not observe any significant protection by the vaccine among those children who were moderately or severely stunted. Similarly, studies in Botswana and Malawi showed protection among well-nourished children, but lower or no protection in undernourished children [11, 12]. However, none of these studies were adequately powered to assess effectiveness in this subgroup of malnourished children, and thus the confidence intervals around these estimates vary widely. It may be worth pooling effectiveness data from African countries to assess this end point with a much larger sample size, which may allow better characterization of vaccine protection among malnourished children. The potential impact of acute diarrhea on the nutritional indicators themselves also needs to be considered.

Genotype-specific vaccine effectiveness was assessed at 2 of the 3 sites. Statistically significant protection was shown against the most common genotype, G1P[8], and lower, insignificant protection against G2P[4] strains owing to the low numbers of G2P[4] strains circulating. Other studies have shown that the monovalent Rotarix vaccine and the pentavalent RotaTeq vaccine exert similar effectiveness against homotypic and heterotypic rotavirus strains in high- and middle-income settings [13]. 

In general, genotype-specific vaccine effectiveness estimates from postintroduction observational studies in Africa have shown protection against the dominant strain in circulation during the time of the study, including both G1P[8] and G2P[4] strains [10–12, 14]. For instance, in Botswana, significant protection was observed against the G2P[4] strain, which was the predominant strain [12]. In many of the country-specific, time-limited studies, the analyses are limited by small sample sizes and not powered to adequately assess strain-specific effectiveness. A pooled analysis of genotype-specific effectiveness using data from the case-control studies conducted in Africa would increase the sample size for each genotype and may give a better assessment than the individual studies [14].

The study by Otieno et al [8], an interrupted time-series analysis, showed significant reductions in rotavirus-specific hospitalizations as well as all-cause diarrheal hospitalizations within the first 3 years of vaccine use. Similar studies have been conducted in several countries worldwide [15], where the incidence of diarrheal hospitalizations before vaccination is compared with that in the postvaccination period. Ecological studies have inherent limitations, including a limited ability to attribute causality, and difficult in adequately controlling for confounders, for example other new interventions or infrastructure improvements during the study period. Although time series analyses provide less robust data, the incidence of rotavirus and all-cause diarrheal hospitalizations declined substantially in Kenyan children after rotavirus vaccine introduction, as seen in similar studies conducted in Africa [16].

The data presented in these 2 analyses support previous studies in Kenya and in other African countries, which showed that rotavirus vaccine has an almost immediate effect on the disease burden, reducing rotavirus-confirmed hospitalizations and all-cause diarrheal hospitalizations within 2–3 years of introduction. Most of these studies have used either of the 2 methods discussed. For example, in other countries with early vaccine introduction (eg, South Africa, Ghana, Malawi, and Rwanda), rotavirus hospitalizations were reduced consistently by approximately 40%–60% in young children <5 years of age. In infants, these reductions were even more dramatic, ranging from 50% to 70% [10–12, 16]. Importantly, reductions in all-cause diarrheal hospitalizations were also observed in approximately 20%–50% of the population.

Several other observations have been recorded in these studies. For instance, the impact on rotavirus-associated diarrheal hospitalizations and all-cause diarrhea hospitalizations has improved year by year after introduction, with improving vaccine coverage [11, 17, 18]. Not surprisingly, vaccine impact is highest in infants, among whom the highest burden of rotavirus disease exists in Africa [19, 20]. There is an outstanding question concerning the duration of protection into the second year of life and beyond. 

Several studies, including that by Khagayi et al [7], have reported subanalyses to assess age-specific vaccine effectiveness in infants <12 months and toddlers 12–24 months of age. Khagayi et al [7] found that there was no significant difference in vaccine effectiveness among children <12 months and those ≥12 months of age, similar to observations from South Africa, Botswana, and Tanzania [7, 10, 12, 21]. However, others have indicated a decreased vaccine impact in the second year of life, presumably associated with waning immunity, including studies in Rwanda, Malawi, and Burkina Faso [18, 22–24]. This question has resulted in studies to evaluate the effect of a “booster” dose of rotavirus vaccine given with measles vaccine at age 9 months, which did boost immune responses in these infants, particularly those with undetectable or very low immune levels [24-26]. Modeled data indicate that this could have an impact on rotavirus-associated deaths in children >12 months of age if first-year efficacy levels are attained [24-26].

This raises the question of indirect or herd protection provided by the vaccination programs. Otieno and colleagues [8] describe the total impact of the vaccine in 2 regions in Kenya, suggesting that herd protection plays a role. Several studies globally have indicated indirect protection in older children who were not age-eligible to receive rotavirus vaccine or who were unvaccinated [26]. In Malawi, herd protection was observed in unvaccinated infants admitted to the hospital with diarrhea [23] and at the population level after programmatic roll-out of the vaccine [6]. Finally, rotavirus vaccine has been demonstrated to offer similar protection against severe rotavirus disease in both human immunodeficiency virus (HIV)–exposed and HIV-unexposed infants, and in HIV-infected infants, although these numbers remain small [10, 11, 23].

These 2 new studies from Kenya [7, 8], combined with the existing data from multiple countries in Africa, highlight the dramatic effect that rotavirus immunization is having on diarrhea-associated mortality and hospitalizations. The evidence is convincing and should encourage the rapid introduction of rotavirus vaccine in countries which have not yet introduced the vaccines. Two new rotavirus vaccines prequalified by the World Health Organization [27] and ongoing vaccine subsidy support from Gavi, The Vaccine Alliance, provide the opportunity for significantly reducing rotavirus mortality and morbidity rates in sub-Saharan Africa and globally.

## Note

***Potential conflicts of interest.*** The authors: No reported conflicts of interest. Both authors have submitted the ICMJE Form for Disclosure of Potential Conflicts of Interest. Conflicts that the editors consider relevant to the content of the manuscript have been disclosed.
